# SLC25A38 as a novel biomarker for metastasis and clinical outcome in uveal melanoma

**DOI:** 10.1038/s41419-022-04718-8

**Published:** 2022-04-11

**Authors:** Zhongyi Fan, Jingjing Duan, Pu Luo, Ling Shao, Qiong Chen, Xiaohua Tan, Lei Zhang, Xiaojie Xu

**Affiliations:** 1 Department of Oncology and Bio-therapeutic Center, Shenzhen Third People’s Hospital, Second Hospital Affiliated to Southern University of Science and Technology, Shenzhen Research Center for Communicable Disease Diagnosis and Treatment, Shenzhen, 518112 China; 2 Department of Oncology, The First Medical Center, General Hospital of PLA, Beijing, 100853 China; 3 Department of Gastrointestinal Oncology, Tianjin Medical University Cancer Institute and Hospital, National Clinical Research Center for Cancer, Tianjin Key Laboratory of Cancer Prevention and Therapy, Tianjin’s Clinical Research Center for Cancer, Tianjin, 300060 China; 4 Department of Ophthalmology, Xuanwu Hospital Attached to the Capital Medical University, Beijing, 100053 China; 5 Department of Genetic Engineering, Beijing Institute of Biotechnology, Beijing, 100850 China

**Keywords:** Eye cancer, Prognostic markers

## Abstract

Risk of metastasis is increased by the presence of chromosome 3 monosomy in uveal melanoma (UM). This study aimed to identify more accurate biomarker for risk of metastasis in UM. A total of 80 patients with UM from TCGA were assigned to two groups based on the metastatic status, and bioinformatic analyses were performed to search for critical genes for risk of metastasis. *SLC25A38*, located on chromosome 3, was the dominant downregulated gene in metastatic UM patients. Low expression of *SLC25A38* was an independent predictive and prognostic factor in UM. The predictive potential of *SLC25A38* expression was superior to that of pervious reported biomarkers in both TCGA cohort and GSE22138 cohort. Subsequently, its role in promoting metastasis was explored in vitro and in vivo. Knock-out of *SLC25A38* could enhance the migration ability of UM cells, and promote distant metastasis in mice models. Through the inhibition of *CBP/HIF*-mediated pathway followed by the suppression of pro-angiogenic factors, *SLC25A38* was situated upstream of metastasis-related pathways, especially angiogenesis. Low expression of *SLC25A38* promotes angiogenesis and metastasis, and identifies increased metastatic risk and worse survival in UM patients. This finding may further improve the accuracy of prognostic prediction for UM.

## Introduction

Uveal melanoma (UM) is the most common primary intraocular malignant tumor in adults [1]. At the time of diagnosis, less than 5% of UM patients had detectable metastatic diseases. However, distant metastases, often to the liver, occur in ~40% of patients during the subsequent course and the mortality rate of metastatic UM is high [1–3]. Therefore, it is particularly important to screen patients with high risk of relapse for subsequent close follow-up and active treatment.

According to the Collaborative Ocular Melanoma Study (COMS) classification, small UM, defined as tumors between 1–2.4 mm in height and 5–16 mm in transverse diameter, only accounts for 3.3% of the patients, while the medium- and large-sized UM accounts for 66.9% and 29.8% of patients, respectively [4]. Small UM tumors usually undergo radiotherapy rather than enucleation; thus, the majority of accessible samples are medium- and large-sized UM. Since UM samples available are mainly medium- or large-sized in clinics, and larger tumors are prone to metastasize, most studies on biomarkers predicting metastasis are conducted in medium- and large-sized tumors [5–8]. Moreover, UM cases in a public database, such as TCGA, are mainly medium- or large-sized. Therefore, we also focused on these tumors to identify more accurate biomarkers for the risk of metastasis in UM.

By far, previous studies have shown that clinicopathological characteristics, chromosomal features, gene mutation, and expression profiles are statistically associated with metastasis in UM [9]. Clinicopathological data, as the most accessible data, can predict increased risk of metastasis [10–14], including male sex, advanced patient age, anterior tumor location, and larger tumor diameter and thickness. However, predictive accuracy of these factors has not been adequate for making personalized clinical decisions. With the development of precise techniques for detecting chromosomal alterations, chromosome 3 loss has shown predictive accuracy superior to clinicopathological features [15]. Risk is also increased by the presence of chromosome 1p or 8p loss, or decreased by the gain of 6p in the tumors [8, 10, 16]. Furthermore, UM can be categorized by 12-gene expression profiling (GEP) into two prognostic subgroups: Class 1 (low metastatic risk) and Class 2 (high metastatic risk). GEP-based assignment of UM has been validated in a prospective multicenter study and is now routinely performed in many centers for clinical use [17, 18]. High expression of PRAME (preferentially expressed antigen in melanoma) was subsequently found to have the potential to identify a group of Class 1 patients with increased metastatic risk [19]. Several genes have been discovered to be frequently mutated in UM, including BRCA1-associated protein-1 (*BAP1*), splicing factor 3B subunit 1 (*SF3B1*), and eukaryotic translation initiation factor 1 A X-chromosomal (*EIF1AX*). Somatic mutations in *BAP1* and *SF3B1* have also been associated with higher metastatic risk, whereas mutations in *EIF1AX* have been shown to be protective [7, 20].

Models with multiple biomarkers always have better prediction results. It was reported that the prognostic value of clinicopathological features can be improved by adding chromosome data, such as the Liverpool Uveal Melanoma Prognosticator Online (LUMPO) [21]. A recent study also found that the combination of the Tumor-Node-Metastasis staging system with chromosome 3 status could provide more accurate predictive information in UM [22]. The success of these models demonstrates the significant role of chromosomal abnormalities in predicting metastasis of UM. Chromosomal abnormalities can directly lead to changes in gene expression levels. However, which genes on chromosome 3 play vital roles in the metastasis of UM is not clear, and the identification of these genes is necessary to elucidate the mechanism of metastasis.

The aim of this study was to identify potentially more accurate biomarker on chromosome 3 responsible for the metastasis of UM and to clarify the mechanism of metastasis. The gene expression data from UM patients in TCGA cohort were deeply analyzed based on the metastatic status. *SLC25A38*, located on chromosome 3, was founded to be the dominant downregulated gene in metastatic UM patients. *SLC25A38* was initially reported to participate in the synthesis of heme in eukaryotes, acting as glycine trans-mitochondrial transporter. The role of *SLC25A38* in anemia has been widely reported [23, 24], but its role in cancer has rarely been studied. Glycine plays a role in iron metabolism, which is crucial to hepatocellular carcinoma progression, thus, representative iron utilization genes including *SLC25A38* were investigated [25]. However, *SLC25A38* does not affect the prognosis of hepatocellular carcinoma patients. Another two recent studies have established the prognosis model for UM from different dimensions [26, 27]. Both models included *SLC25A38*, indicating the important effects of *SLC25A38* on UM progression. Since they are the study of the prognosis model, the mechanism of *SLC25A38* has not been discussed in detail.

In our study, the predictive potential of *SLC25A38* expression was validated in two independent datasets and the detailed mechanism of *SLC25A38* was explored. This finding may further improve the accuracy of prognostic prediction for UM.

## Methods

### Clinical cohort and study design

A total of 80 UM patients with mRNA expression profiling were included from The Cancer Genome Atlas (TCGA) database. Baseline clinicopathological information, metastatic status, and final clinical outcome were recorded for each patient. UM patients were divided into two subgroups based on their metastatic status, and bioinformatic analyses were performed to screen the key genes on chromosome 3 relevant to metastasis. Another cohort with 63 UM patients (GSE22138) were chosen from the GEO database. The predictive accuracy of SLC25A38 expression for risk of metastasis was compared to that of previously reported biomarkers (including chromosome 3 status, mutation of *BAP1* and GEP class) in both TCGA cohort and GSE22138 cohort.

After bioinformatic analyses on TCGA UM samples and GSE22138 UM cases, the role of SLC25A38 in UM metastasis were predicted and then validated in vitro and in vivo. Furthermore, 28 UM FFPE specimens from General Hospital of PLA were included to examine the correlation of SLC25A38 expression and angiogenesis-related molecules expression. This study was approved by the Institutional Review Committee of the Chinese PLA General Hospital, and all patients received the informed consent.

### Cell lines and culture

Human UM cell lines (OCM-1, MUM-2B, and 92-1) were purchased from American Type Culture Collection (ATCC). Cells were cultured accordingly. UM cells with *SLC25A38* knock-out were constructed using CRISPR/CAS9 technique. The primers used were as follows: KO#1: sg-GCAGTTACATCCGGTGATCAAGG, KO#2: sg-TGATCACCGGATGTAACTGCAGG.

### Protein extraction and western blot

Cells were fully lysed in RIPA buffer. Western blot analysis was performed. Briefly, total proteins were separated on SDS-PAGE gels and then transferred to PVDF membrane. After blocking, the membrane was incubated overnight at 4 °C with primary anti-SLC25A38 (1:200, Abcam, ab133614) or anti-HIF1α (1:500, Proteintech, 20960-1-AP). The next day, secondary antibodies were used accordingly and the protein bands were visualized. Anti-β-Actin (1:2,000, Santa Cruz, sc-47778) was used as a control.

### RNA isolation and RT-PCR

Total mRNA was extracted using RNA Extraction Kit (Takara Bio Inc.) according to the manufacturer’s instructions. RNA was reverse transcribed into cDNA by PrimeScript™ RT reagent Kit (Takara). Real-time PCR was performed using SYBR Premix Ex Taq™ (Takara). The mRNA levels of SLC25A38 were normalized to β-Actin. The primers used are listed in Table S1.

### Cell migration and invasion assay

Wound healing assay and transwell assay were used to evaluate cell migration and invasion. When the cells were attached as confluent monolayers, a wound-healing assay was performed. UM cells grown in 6-well plates were mechanically scratched using a 10 μL pipette tip to create a linear region. Cells were then washed with PBS to remove the debris and were cultured in the medium with 1% FBS for 48 h to allow wound healing. For transwell invasion assay, chambers (Corning Inc.) with 8-μm pore size polycarbonate membrane were used. Cells were seeded in the upper chambers with serum-free medium, while a medium containing 10% FBS was placed in the lower well as a chemoattractant. Twenty-four hours later, cells that invaded through the membrane were fixed with 4% paraformaldehyde and stained with crystal violet.

### Vascular ring formation of HUVEC cells

Briefly, 100 μL of Matrigel was added to each well of a 24-well plate and polymerized at 37 °C for 30 min. After co-culture with different UM cells, equal amounts of HUVEC cells (1 × 10^5^ cells/well) re-suspended in FBS-free DMEM medium were seeded in each well. Then the cells were examined to assess the formation of capillary-like structures 6 h later. And the number of tubes and normalized tube length were scanned and quantified in at least five low-power fields (200×).

### Immunofluorescence (IF)

OPAL-4-plex reagents (Perkin-Elmer) were used for multi-color IF according to instructions. Images were acquired on the Vectra Automated Quantitative Pathology Imaging System (Perkin-Elmer), and analyzed using Inform software (Perkin-Elmer) for the SLC25A38, CBP, and CD31 expression rate in the entire tissue sections. Anti-SLC25A38 (Abcam, ab133614), anti-CBP (Santa Cruz, sc-365387) and anti-CD31 (Abcam, ab134168) were used at dilutions of 1:100, 1:100 and 1:1000, respectively.

### Tumor xenografts in vivo

Animal studies were performed in accordance with protocols approved by the Institutional Animal Care and Use Committee of Chinese PLA General Hospital. BALB/c nu/nu mice (6-week-old, male) were randomly divided into two groups (*n* = 5 per group). The indicated cells (1 × 10^6^ cells) were injected subcutaneously into the inguinal folds of mice at random. Tumor sizes were measured at the indicated times, and tumor volumes were estimated. The mice were anesthetized and killed 35 days after inoculation, and the mice were then anatomized and photographed.

For the metastasis mice model, 1 × 10^6^ OCM-1 cells with knock-out of SLC25A38 or equal amounts of control cells were injected intravenously via the lateral tail vein of NOD-SCID mice (6-week-old, male; *n* = 8/group). All mice were maintained for ~50 days until the micro-PET examination.

### PET imaging of glucose uptake in mice

PET imaging of mice was performed using an animal PET scanner (Philips Corp.) according to indications. After anesthetization with pentobarbital, mice were injected intravenously with 3.7 MBq (100 μCi) of ^18^F radio-labeled fluorodeoxyglucose (^18^FDG). A 5-min emission scan was performed to obtain attenuation correction data in the prone position at 60 min after injection, and a 10-min delayed scan was acquired at 2 h. For each mouse, the radioactivity was calibrated according to the known limits of the injected tracer and presented as a percentage of the tissue dose.

### Bioinformatic and statistical analyses

The expression of SLC25A38 or BAP1 was recorded as a dichotomous (high vs. low) variable by the optimal cut-off value using *Z*-score. The conventional differential gene analysis, GO analysis, and KEGG analysis, together with the presentation of heatmap and volcano graph were performed by R software. For gene set enrichment analysis (GSEA), SLC25A38 expression was treated as a numeric variable. A continuous-type cls file of the SLC25A38 profile was applied to phenotype labels in GSEA. The metric for ranking genes in GSEA was set as ‘Pearson’, and the other parameters were set to their default values. GSEA was performed using GSEA 2.2.3 software.

All statistical analyses were performed using SPSS Statistics. All data were representative of at least three independent experiments and illustrated as the means ± standard deviation (SD). The Student’s *t*-test or χ^2^-test was used for two-group comparisons. Survival curves according to SLC25A38 expression were estimated with the Kaplan–Meier method and a log-rank test was used to assess significance. Univariate and multivariate analyses were performed using Cox regression analysis. The logistic model was used to analyze factors related to tumor metastasis. All statistical tests were two-sided. A *P*-value < 0.05 was considered as statistical significance.

## Results

### SLC25A38 expression correlates to metastatic status in TCGA UM cohort

Based on the metastasis status, UM patients from TCGA (*n* = 80) were divided into two groups (54 patients without metastasis, and 26 patients with metastasis). Clinical, histological features and chromosomal data were compared between the two groups (Table S2). Metastatic tumors tended to have larger diameter (*P* = 0.0408) and thickness (*P* = 0.0495). The frequency of chromosome 3 loss (*P* = 0.016) and chromosome 8q gain (*P* = 0.038) were higher in metastatic cases, which is in accordance with previous results [15]. As Table S3 shows, older age, longer tumor basal diameter, together with chromosome 3 loss and chromosome 8q gain correlated to poor clinical outcomes. After adjusting by age, only chromosome 3 status tended to predict overall survival (*P* = 0.082). Chromosome 3 loss have been shown to provide more accurate metastatic risk [22], therefore, we focused on the functional genes on chromosome 3.

**Fig. 1:**
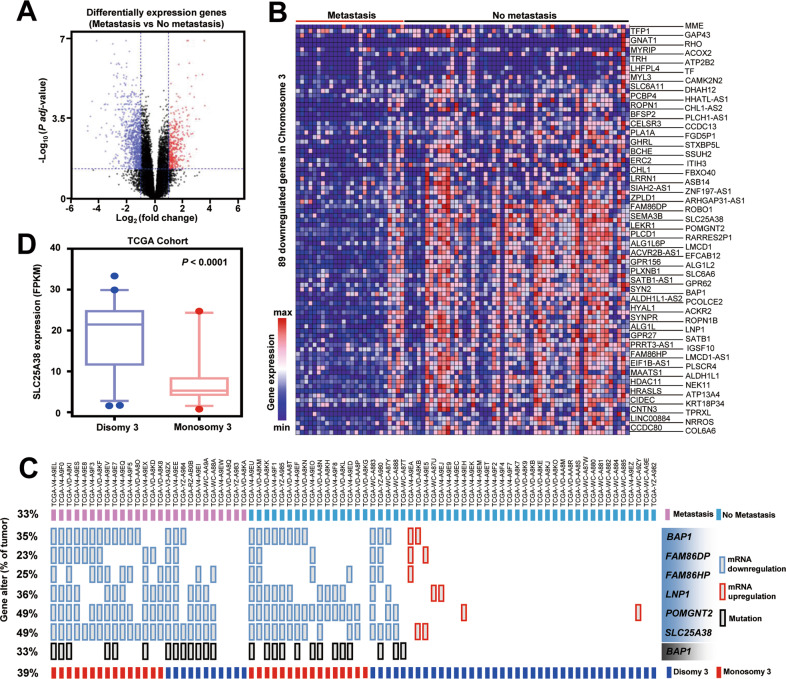
Gene expression profiling of primary uveal melanomas (UMs) based on metastatic status. **A** Volcano plots showing differentially expression genes between metastatic tumors and non-metastatic tumors in TCGA UM cohort. **B** Heatmap showing the 89 most highly downregulated genes located on chromosome 3. Blue: decreased gene expression, red: increased gene expression. **C** Molecular landscape in 80 primary UMs of TCGA cohort. Mutation or expression status for six genes, the metastatic status of tumor sample, and chromosome 3 copy number alterations are indicated. **D** SLC25A38 was highly expressed in tumors with disomy 3 in TCGA UM cohort.

Genes that were differentially expressed between the two groups (metastasis vs. no metastasis) were shown in Fig. 1A and Fig. S1. There were 307 upregulated genes and 714 downregulated genes in metastatic UM tumors (*P* adj < 0.05, fold change > 2). Among them, 89 downregulated genes on chromosome 3 were screened out (Fig. 1B). In order to identify more universal biomarkers among the 89 genes, we found out six downregulated genes that appeared in >20% of patients, that is *BAP1, FAM86DP, FAM86HP, LNP1, POMGNT2*, and *SLC25A38* (Fig. S2). The variations of these 6 genes in each patient of TCGA cohort were shown in Fig. 1C. Nearly half of UM cases had downregulated POMGNT2 and SLC25A38; however, the frequency of SLC25A38 downregulation (85%, 22/26) in metastatic UM was a little higher than that of POMGNT2 downregulation (73%, 19/26), indicating that SLC25A38 might play more critical roles in the metastasis of UM. The mutation of *BAP1* was previously reported to be associated with the UM metastasis, but only 50% (13/26) of metastatic tumors presented with *BAP1* mutation, whose incidence was also much lower than that of SLC25A38 downregulation. The Logistic Regression and Cox analyses were then conducted to identify independent predictive and prognostic biomarkers among these genes, respectively. As Table 1 and Table S4 shown, the low expression of these 6 genes, together with *BAP1* mutation and chromosome 3 loss were all related to metastasis status and poor survival; however, only SLC25A38 downregulation could predict metastasis and poor clinical outcome after multivariate analysis; thus, SLC25A38 was selected for further investigation. And the expression level of SLC25A38 did decrease in cases with chromosome 3 monosomy in TCGA UM cohort (Fig. 1D).

**Table 1:** Univariate and multivariate analysis for risk of metastasis in patients with UM by logistic regression analysis from TCGA dataset.

Variable	Univariate logistic regression		Multivariate logistic regression	
	HR (95%CI)	*P*-value	HR (95%CI)	*P*-value
**BAP1 Low expression**				
No	1.00		1.00	
Yes	5.331(1.919–14.81)	**0.001**	0.442(0.076–2.582)	0.364
**FAM86DP Low expression**				
No	1.00		1.00	
Yes	7.187(2.154–23.98)	**0.001**	3.683(0.730–18.58)	0.114
**FAM86HP Low expression**				
No	1.00		1.00	
Yes	4.217(1.430–12.43)	**0.009**	1.220(0.276–5.395)	0.793
**LNP1 Low expression**				
No	1.00		1.00	
Yes	4.773(1.741–13.08)	**0.002**	2.666(0.482–14.74)	0.261
**POMGNT2 Low expression**				
No	1.00		1.00	
Yes	5.429(1.929–15.28)	**0.001**	0.118(0.006–2.232)	0.154
**SLC25A38 Low expression**				
No	1.00		1.00	
Yes	14.30(4.220–48.46)	**1.9** **×** **10**^**−5**^	77.30(3.645–1638)	**0.005**
**BAP1 Mutation**				
No	1.00		1.00	
Yes	3.154(1.172–8.488)	**0.023**	0.684(0.151–3.109)	0.623
**Chromosome 3 loss**				
No	1.00		1.00	
Yes	3.239(1.224–8.568)	**0.018**	1.419(0.268–7.519)	0.681

The bold *P*-value indicates statistical significance.

In addition, we found that the expression level of SLC25A38 was negatively correlated with that of biomarkers related to cell proliferation, such as Ki-67 and CCNB1 (Fig. S3A, B) in TCGA UM specimens, demonstrating a role of SLC25A38 in the regulation of UM growth. Although the expression level of SLC25A38 did not reach significant difference among tumors with different T stages, the median value of SLC25A38 expression showed a trend of decreasing level of expression with increasing T stage (Fig. S3C).

### The predictive accuracy of SLC25A38 expression is superior to that of others for predicting risk of metastasis in UM

**Fig. 2:**
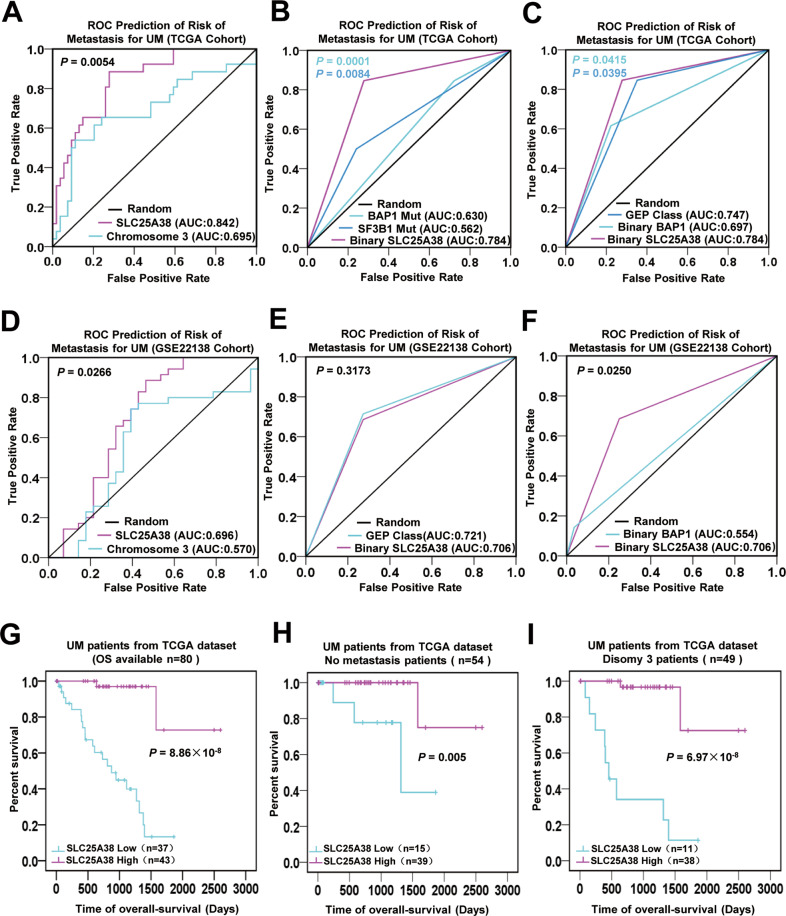
Model accuracy and survival analyses in UM. **A–F** Receiver Operator Curves based on different feature sets in TCGA cohort (**A**–**C**) and GSE22138 cohort (**D**–**F**). The predictive accuracy of SLC25A38 expression for risk of metastasis was superior to that of other biomarkers. The *P*-value in light blue in **B** indicates Binary SLC25A38 vs *BAP1* Mut, and the *P*-value in dark blue in **B** indicates Binary SLC25A38 vs *SF3B1* Mut. The *P*-value in light blue in **C** indicates Binary SLC25A38 vs Binary BAP1, and the *P*-value in dark blue in **C** indicates Binary SLC25A38 vs GEP Class. **G**–**I** Survival curves based on SLC25A38 expression level. Low expression of SLC25A38 predicted poor clinical outcomes not only in all patients but also in patients without metastasis or patients with disomy 3 in TCGA UM cohort. AUC Area Under Curve.

We next explored whether or not a low expression of SLC25A38 can be a predictive biomarker for the risk of metastasis in UM. By using ROC curves, the predictive accuracy of SLC25A38 expression was compared to that of previous widely reported biomarkers, including chromosome 3 status, BAP1 mutation, SF3B1 mutation, and GEP class. When SLC25A38 expression was regarded as a continuous variable, its predictive potential (0.842) is much higher than that of chromosome 3 loss (0.695) in TCGA UM patients (Fig. 2A, *P* = 0.0054). Then the expression of SLC25A38 was recorded as a dichotomous (high vs. low) variable by the optimal cut-off value using *Z*-score. The *BAP1* mutation status and *SF3B1* mutation status only had an accuracy of 0.630 and 0.562 for predicting risk of metastasis, respectively; whereas binary SLC25A38 expression increased its accuracy to 0.784 (Fig. 2B, Binary SLC25A38 vs *BAP1* Mut, *P* = 0.0001; Binary SLC25A38 vs *SF3B1* Mut, *P* = 0.0084). In addition, both the predictive accuracy of GEP class (0.747) and that of binary BAP1 expression (0.697) was inferior to that of binary SLC25A38 expression (0.784), indicating the excellent predictive power of SLC25A38 expression for the risk of metastasis (Fig. 2C, Binary SLC25A38 vs Binary BAP1, *P* = 0.0415; Binary SLC25A38 vs GEP Class, *P* = 0.0395).

Another UM cohort (*n* = 63) with mRNA expression profiling from GSE22138 was used to further investigate the predictive role of SLC25A38 expression. As shown in Fig. 2D–F, we drew nearly the same conclusion from GSE22138 cohort with TCGA cohort (continuous SLC25A38 vs chromosome 3, *P* = 0.0266; Binary SLC25A38 vs Binary BAP1, *P* = 0.0250), except that the accuracy of GEP class (0.721) was a little higher than that of SLC25A38 level (0.706), which did not reach statistical difference (*P* = 0.3173). As Fig. S4A shown, although binary SLC25A38 expression had an accuracy of 0.739 which was a little superior to that of GEP class (0.721) when the samples of the two cohorts are merged together, it reached no statistical difference (*P* = 0.2356). These results showed that the predictive accuracy of SLC25A38 was superior to that of previously reported biomarkers including chromosome 3 status and *BAP1* status, and was at least equivalent to that of the widely used GEP class.

### Low expression of SLC25A38 is an independent prognostic biomarker in UM

Survival curves according to SLC25A38 expression showed that low expression of SLC25A38 correlated to poor clinical outcome not only in all patients but also in patients without metastasis (Fig. 2G, H). Although low expression of SLC25A38 mostly appeared in patients with chromosome 3 loss, it also indicated shorter overall survival in cases with chromosome 3 disomy (Fig. 2I). As shown in Table S5, multivariate Cox analyses showed that SLC25A38 expression was an independent prognostic biomarker in UM patients after adjustment by clinicopathological parameters. Moreover, low expression of SLC25A38 is also associated with risks of metastasis development (Fig. S4B). The above results demonstrated that as a tumor suppressor, low expression of SLC25A38 was an indicator of higher risk for metastasis and worse clinical outcome in UM patients.

### SLC25A38 is situated upstream of metastasis-related pathways

**Fig. 3:**
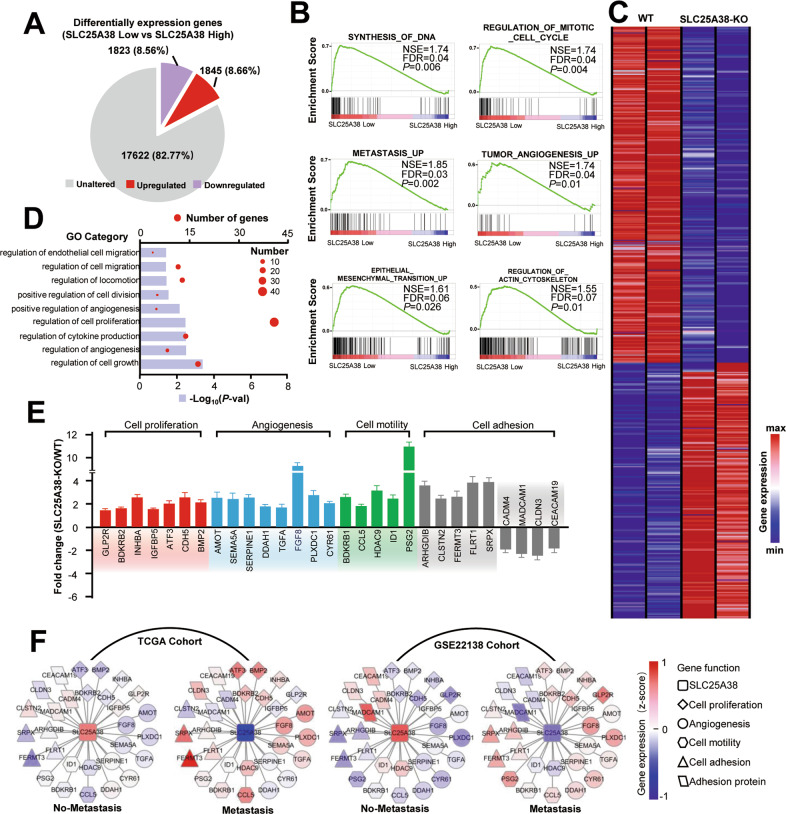
SLC25A38 is the upstream of several metastasis-related pathways. **A** Pie chart illustrating differentially expression genes between SLC25A38-low tumors and SLC25A38-high tumors in TCGA UM RNA-seq dataset. **B** GSEA showing that SLC25A38 expression was negatively correlated with proliferative and metastatic gene signatures in the TCGA UM dataset. **C** Heatmap showing differentially expression genes in UM cells with SLC25A38 knock-out relative to control cells. **D** The most significant differentially GO items upon SLC25A38 knock-out. **E** Differentially expression genes in the SLC25A38-silenced UM cells in the indicated signaling pathways analyzed by qRT-PCR. **F** These series of networks demonstrated the changes in SLC25A38 and its neighboring genes (involving in cell proliferation, cell adhesion, cell motility, and angiogenesis) in terms of expression during metastasis in TCGA UM specimens and GSE22138 tumors.

To give a better understanding of the SLC25A38-involved pathway in UM, differentially expressed genes between the SLC25A38-high group and SLC25A38-low group in TCGA cohort were analyzed. As shown in Fig. 3A, compared to SLC25A38-high cases, 1823 genes were upregulated and 1845 genes were downregulated in SLC25A38-low tumors (*P* adj < 0.05, fold change > 2). The results of GSEA showed that SLC25A38 not only can regulate cell proliferation process including DNA synthesis and mitotic cell cycle, but also is closely related to various biological behaviors related to tumor metastasis, such as the regulation of cytoskeleton protein, tumor angiogenesis, and epithelial–mesenchymal transformation (Fig. 3B). Furthermore, we performed RNA sequence on UM cells with or without SLC25A38 knock-out. As expected, knock-out of SLC25A38 lead to great changes in cell proliferation, adhesion, migration, and vascular growth (*P* adj < 0.05, fold change > 1.5; Fig. 3C, D), which coincided with the bioinformatic results of tumors from TCGA cohort. The validation of PCR also demonstrated an upregulated level of molecules related to cell growth, adhesion, and angiogenesis upon knock-out of SLC25A38 (Fig. 3E). Further analyses showed that metastatic cases tended to have lower SLC25A38 expression and higher levels of molecules involving in cell proliferation, cell adhesion, cell motility, and angiogenesis compared to non-metastatic tumors in both TCGA cohort and GSE22138 cohort (Fig. 3F). The above evidences indicated that SLC25A38 is situated upstream of metastasis-related pathways.

### Knock-out of SLC25A38 promotes the metastasis and angiogenesis in UM

**Fig. 4:**
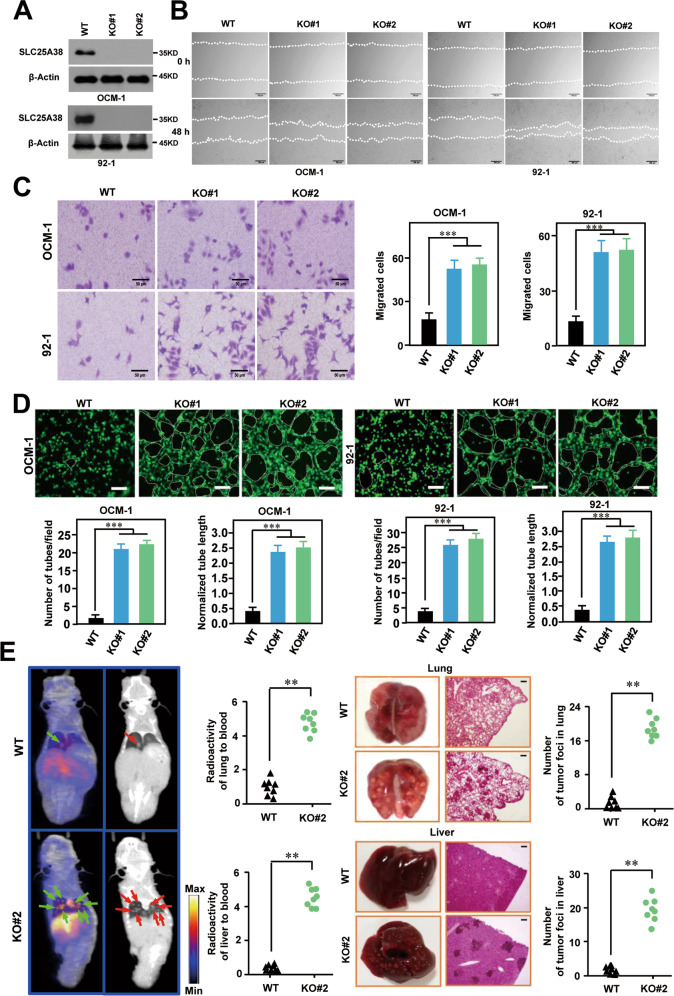
Silencing SLC25A38 promotes the malignant properties of UM cells in vitro and in vivo. **A** UM cells with SLC25A38 knock-out were successfully constructed. **B**, **C** Wound healing assay (**B**) and transwell invasion assay (**C**) showing that SLC25A38 knock-out could enhance the migration and invasion ability of UM cells. **D** Vascular ring formation analyses indicate the increasing ability of angiogenesis upon SLC25A38 knock-out. The number of tubes was counted and normalized tube length was calculated, scale bar 100 μm. **E** PET imaging and H&E staining showed that mice inoculated with SLC25A38 knock-out UM cells had more lung metastases and live metastases compared to that injected with control UM cells, scale bar 100 μm.

Given that *GNAQ/11* was the most frequent mutation in UM and closely related to the prognosis of UM, we found that the expression level of SLC25A38 was significantly different between different *GNAQ* mutation status but not *GNA11* (Fig. S5A, B). To further clarify the definite independent role of SLC25A38 in UM, two types of cell lines OCM-1 (*GNAQ*^*WT*^), MUM-2B (*GNAQ*^*WT*^), and 92-1(*GNAQ*^*Q209L*^) were selected to perform in vitro and in vivo experiments. As shown in Fig. 4A and Fig. S6A, UM cells with SLC25A38 knock-out were successfully constructed. CCK8 assay showed that SLC25A38 knock-out led to the increasing proliferation of UM cells (Fig. S6A). 6-week-old BALB/c nu/nu mice were used for constructing subcutaneous xenograft models. Tumors with SLC25A38 knock-out grew larger and faster in vivo (Fig. S6B), suggesting that SLC25A38 acts as a tumor suppressor in UM and its dramatic downregulation contributes to a faster rate of proliferation in cancer cells.

Effects of SLC25A38 on cell migration in vitro were then evaluated by wound healing assay (Fig. 4B and Fig. S7A) and transwell assay (Fig. 4C and Fig. S7B, C). As expected, both the two approaches demonstrated that UM cells with knock-out of SLC25 A38 showed a higher ratio in migration. In addition, SLC25A38 knock-out in UM cells resulted in increased ring formation of the HUVEC cells, indicating the increasing ability of angiogenesis upon SLC25A38 knock-out (Fig. 4D and Fig. S7D, E). Next, OCM-1 cells with SLC25A38 knock-out or equal amounts of control cells were injected intravenously via the lateral tail vein of NOD-SCID mice to construct metastasis models. As shown in Fig. 4E, PET imaging of mice indicated that UM cells with downregulated SLC25A38 had stronger ability of proliferation and metastasis in blood, and can form more lung and liver metastases. Therefore, the fact that downregulated SLC25A38 could promote the metastasis and progression of UM was verified both in vitro and in vivo.

### Downregulated SLC25A38 boosts angiogenesis via enhancing release of pro-angiogenic factors

**Fig. 5:**
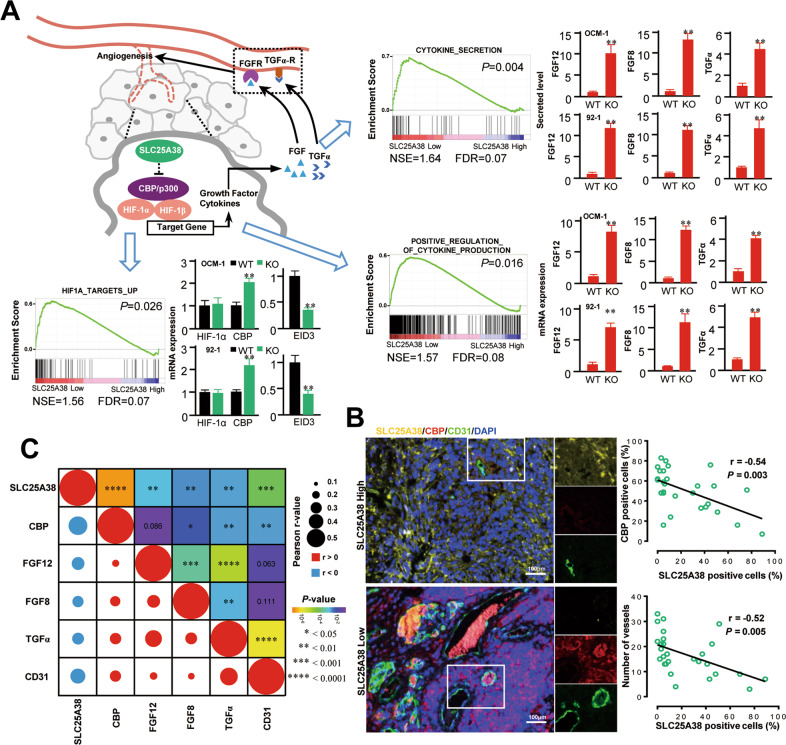
Downregulated SLC25A38 boosts angiogenesis. **A** A proposed model underlying the role of SLC25A38 in UM angiogenesis. Through the promotion of the coactivator (CBP/p300) of HIF upon SLC25A38 knock-out, its target genes including pro-angiogenic factors are overexpressed and thus promote angiogenesis. GSEA of TCGA cohort and PCR validation of UM cells verified this proposal. **B** Correlation between SLC25A38 and CBP or CD31 protein expression in UM specimens. Tumor sections from 28 UM specimens were immunofluorescence stained with anti-SLC25A38, anti-CBP, and anti-CD31 antibodies. Left: representative images. Right: the correlation between SLC25A38 and CBP or CD31 protein expression was calculated by Pearson correlation coefficient. **C** Correlation between SLC25A38, CBP, FGF8, FGF12, TGFα, and CD31 expression in TCGA UM RNA-seq dataset. The Pearson correlation coefficient and *P*-value are indicated.

Angiogenesis is closely related to tumor growth and metastasis. Only with angiogenesis can the tumor grow larger and eventually metastasize. Based on the above bioinformatic and experimental results, we focused on the role of SLC25A38 in inhibiting angiogenesis. As shown in Fig. 5A, GSEA results of TCGA UM tumors showed that SLC25A38 expression level negatively correlated to that of HIF-related pathway targets and the production and secretion of pro-angiogenic factors. Subsequent PCR of UM cells (OCM-1 and 92-1) validated that as a transcriptional coactivator for HIF1α, CBP was upregulated upon SLC25A38 knock-out. And the level of EID3 which can inhibit the activities of p300 (also acts as a transcriptional coactivator for HIF) decreased in SLC25A38 knock-out UM cells. However, no correlation was found between SLC25A38 and HIF-1α in mRNA level. The increased level of CBP and the decreased level of EID3 lead to the increased transcriptional activity of HIF signaling pathway, followed by overexpressed targets and excessive secretion of pro-angiogenic cytokines, such as FGF12, TGF8, and TGFα. As known, HIF-1α expression has important post-transcriptional mechanisms. In protein level we found SLC25A38 knock-out enhanced the protein level of HIF-1α in UM cells under both conditions of hypoxia or normoxia (Fig. S8A, B).

To further explore the relationship between SLC25A38 and CBP/HIF-related angiogenesis, 28 UM specimens were included from General Hospital of PLA. As shown in Fig. 5B, CBP were overexpressed and more CD31-positive neovascularization occurred in UM cases with downregulated SLC25A38, demonstrating the inhibitory role of SLC25A38 in angiogenesis. In accordance with the results of our UM specimens, SLC25A38 expression level negatively correlated to that of CBP, that of pro-angiogenic cytokines (FGF12, TGF8, and TGFα) and that of CD31(indicates neovascularization) in TCGA cohort (Fig. 5C). In summary, our results demonstrated that downregulated SLC25A38 can promote the transcription of HIF-related pathway target genes via the relief of CBP inhibition, thus lead to the excessive production and secretion of pro-angiogenic factors and eventually boost angiogenesis in UM.

## Discussion

UM is a potentially lethal cancer, but the factors that determine the unfavorable course of this disease remain unclear. Large UM requires intense treatment; however, unreasonable therapy has a negative impact on vision and the quality of life. Decisions on the treatment need to precede the occurrence of fatal metastasis. Consideration of clinical, histopathological, and cytogenetic prognostic factors contributes to determining the different prognosis groups of UM patients. Our study identified a novel biomarker, SLC25A38, for predicting metastasis and clinical outcome in UM patients. The predictive accuracy of SLC25A38 was superior to that of previous reported predictive biomarkers such as the well-known chromosome 3 loss and BAP1 mutation and was equivalent to that of the widely used 12-gene GEP class. SLC25A38 was then verified to be situated upstream of metastasis-related pathways and play crucial roles in angiogenesis in UM, which increased our understanding of UM metastasis.

As a member that belongs to the mitochondrial solute carrier family, SLC25A38 was initially reported to participate in the synthesis of heme in eukaryotes [28]. It may act by transducing glycine into mitochondria, or as a transporter of glycine/5-aminolevulinic acid (ALA) across mitochondrial inner membrane. Glycine/ALA trans-mitochondrial transport is a key and rate-limiting step in the biosynthesis of heme, which is essential in diverse biological processes such as respiration, detoxification, and signal transduction [29, 30]. The role of *SLC25A38* in anemia has been widely reported, but its role in cancer has rarely been studied. A previous study showed that the increased mitochondrial glycine metabolism is strongly correlated with rates of proliferation across cancer cells [31]. Since SLC25A38 acts as a transporter of glycine across mitochondrial inner membrane, we hypothesize that knock-down of SLC25A38 may inhibit the transport of glycine to cytoplasm, and the accumulation of glycine in mitochondria may lead to the increase of glycine metabolism, thus facilitate the maintenance of the malignant properties of cancer cells. In our study, SLC25A38 was screened out in an unsupervised manner from a global genomic database and its predictive value was independently validated in two cohorts. We verified the positive effects of SLC25A38 knock-out on UM growth. Moreover, we identified that SLC25A38 could control UM metastasis through the regulation of angiogenesis in vitro and in vivo. It is the first time to deeply explore the role of SLC25A38 in UM and is an important supplement of the mechanisms of UM metastasis.

UM usually metastasizes through hematogenous spread; thus, it is particularly important to elucidate the mechanism of angiogenesis in UM. HIF-induced upregulation of VEGF and ANGPTL4 promote the angiogenic phenotype in UM [32]. FGF can enhance the metastasis of UM cells via store-operated calcium entry [33]. Our research also verifies that HIF-related pathway followed by excessive production of downstream cytokines including FGF boost angiogenesis in UM, which is in accordance with previous results. In a pre-clinical study, bevacizumab has been reported to inhibit hepatic micro-metastasis of UM cells [34]. And clinical studies have shown that anti-angiogenic treatment can lead to the reduced central foveal thickness and visual improvement in some UM patients [35]. As a promising therapeutic target for metastatic UM, MLN4924 treatment can disturb the paracrine secretion of NF-kB-mediated VEGF-C and its dependent angiogenesis [36]. The inhibitory effects of SLC25A38 on angiogenesis we found were based on the suppression of transcriptional coactivator for HIF signaling pathway and HIF-1α protein level, and to our best knowledge, it is the first time to confirm the relationship between SLC25A38 and CBP/HIF-related tumor growth and metastasis. However, few studies have demonstrated the important role of glycine metabolism in HIF-related disease. The dysregulation of serine/glycine metabolism might lead to a lack of amino acids, followed by an elevated level of ROS, resulting in increased HIF-1α in colorectal cancer [37]. Glycine decarboxylase and HIF-1α expression were verified to be negative prognostic factors in primary resected early-stage non-small cell lung cancer [38]. Charandeep Singh et al. reported that serine and 1-carbon metabolism were required for HIF-mediated protection against retinopathy of prematurity [39]. Since SLC25A38 acts as a glycine trans-mitochondrial transporter, it is likely to affect the function of HIF-related pathways. Actually, we found that SLC25A38 could promote the transcription of HIF-related pathways target genes via the relief of its transcriptional coactivator CBP, which broaden our views about the relationship between SLC25A38 or glycine metabolism and HIF-related cancer progression. However, the deeper mechanisms underlying how SLC25A38 inhibits transcriptional coactivators remain unknown. Further investigations will focus on this project and may clue to the anti-angiogenesis therapy in UM.

The clinical, histopathological, and cytogenetic features related to UM metastasis and prognosis were comprehensively reviewed before [9]. We compared the predictive potential of SLC25A38 for risk of metastasis with that of chromosomal features and identified gene expression groups. The results showed that SLC25A38 had an apparent advantage in forecasting metastasis. By far, the 12-GEP-based classification of UM has been validated and widely used in clinic, and can provide more accurate predictive information than clinicopathological or chromosomal characteristics. Using ROC curves, the predictive potential of SLC25A38 (AUC: 0.784) was superior to that of GEP class (AUC: 0.747, *P* = 0.0396) in TCGA cohort. However, there was no statistical difference between the predictive accuracy of SLC25A38 (AUC: 0.706) and that of GEP class (AUC: 0.721, *P* = 0.3173) in GSE22138 cohort. Since the sample size of both cohorts is relatively small, the inconsistency may result from sample bias. Despite that, our results showed that the predictive accuracy of SCL25A38 expression is at least equivalent to that of the widely used 12-gene GEP class. Undoubtedly, the detection of one gene is simpler than that of 12-gene, so it might have greater application prospects if the potential of SCL25A38 expression can be confirmed in larger cohorts. In addition, the optimal cut-off value used to distinguish SLC25A38 from high expression and low expression also need to be investigated, which will benefit future clinical work.

To conclude, we found that SLC25A38 was a novel biomarker for metastasis and survival for UM. Through the suppression of transcriptional coactivator for HIF signaling pathway, SLC25A38 was suited upstream of HIF signaling pathway and inhibited the production of pro-angiogenic factors, thus impeded angiogenesis and tumor progression. Detection of SLC25A38 expression alone can provide more accurate predictive information than previous predictive biomarkers, which we believe is much simpler and easier to popularize in clinical practice. Therefore, our findings may greatly improve the accuracy and convenience of prognostic prediction for UM.

## Supplementary information

Supplemental Material

aj-checklist

## Data Availability

All data generated or analyzed during this study are included in this published article and its supplementary information files.
