# Supplementary material for: SLC25A38 as a novel biomarker for metastasis and clinical outcome in uveal melanoma
Source: Cell Death Dis. 2022 Apr 11;13(4):330. doi: 10.1038/s41419-022-04718-8 (PMC9001737; doi:10.1038/s41419-022-04718-8)
Supplement: Supplementary file 1 — Supplemental Material [file 41419_2022_4718_MOESM1_ESM.pdf]

Figure S1

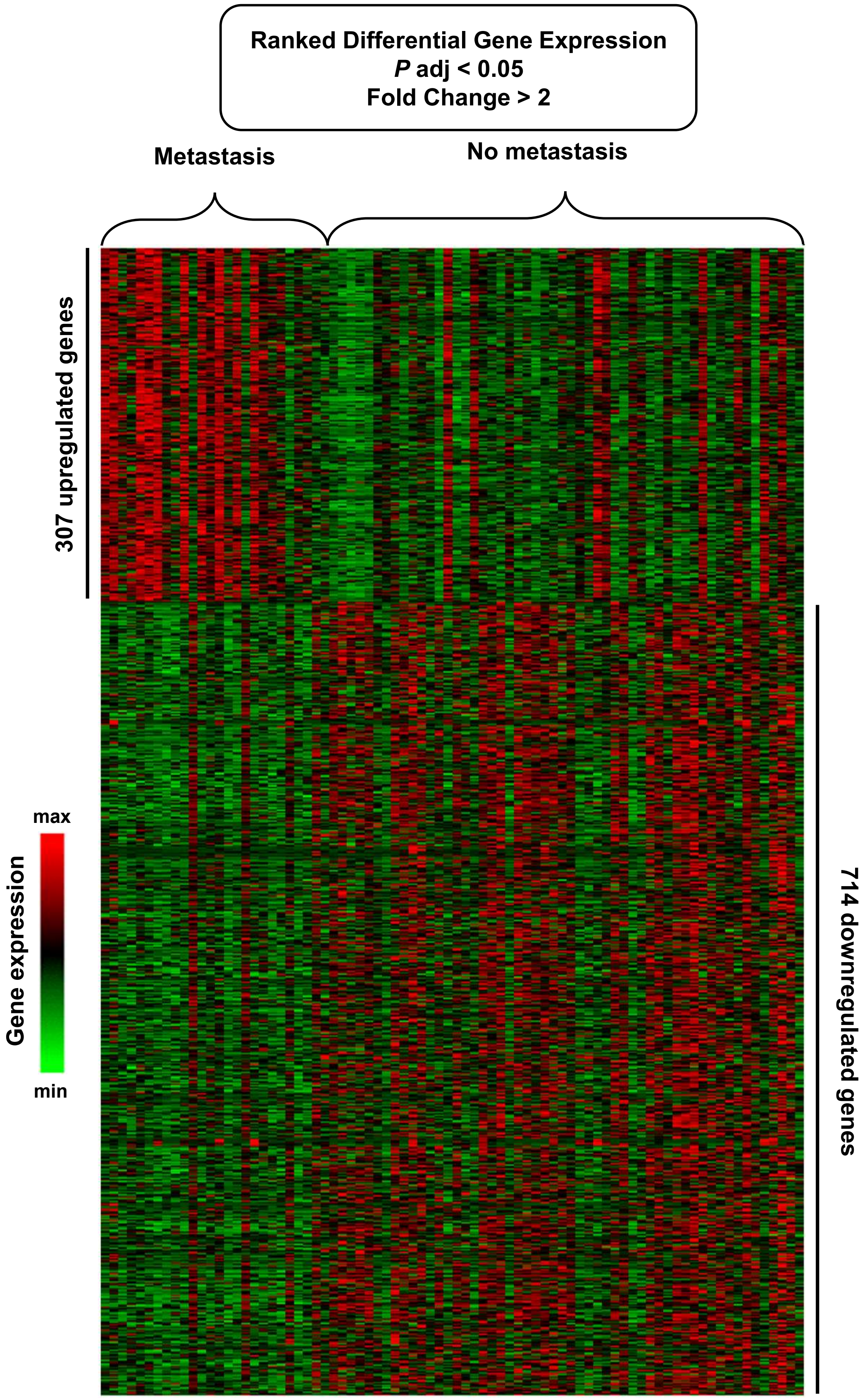

Figure S1. Heatmap showing differentially expression genes between metastatic tumors and non-metastatic tumors in TCGA UM data set.

Figure S2

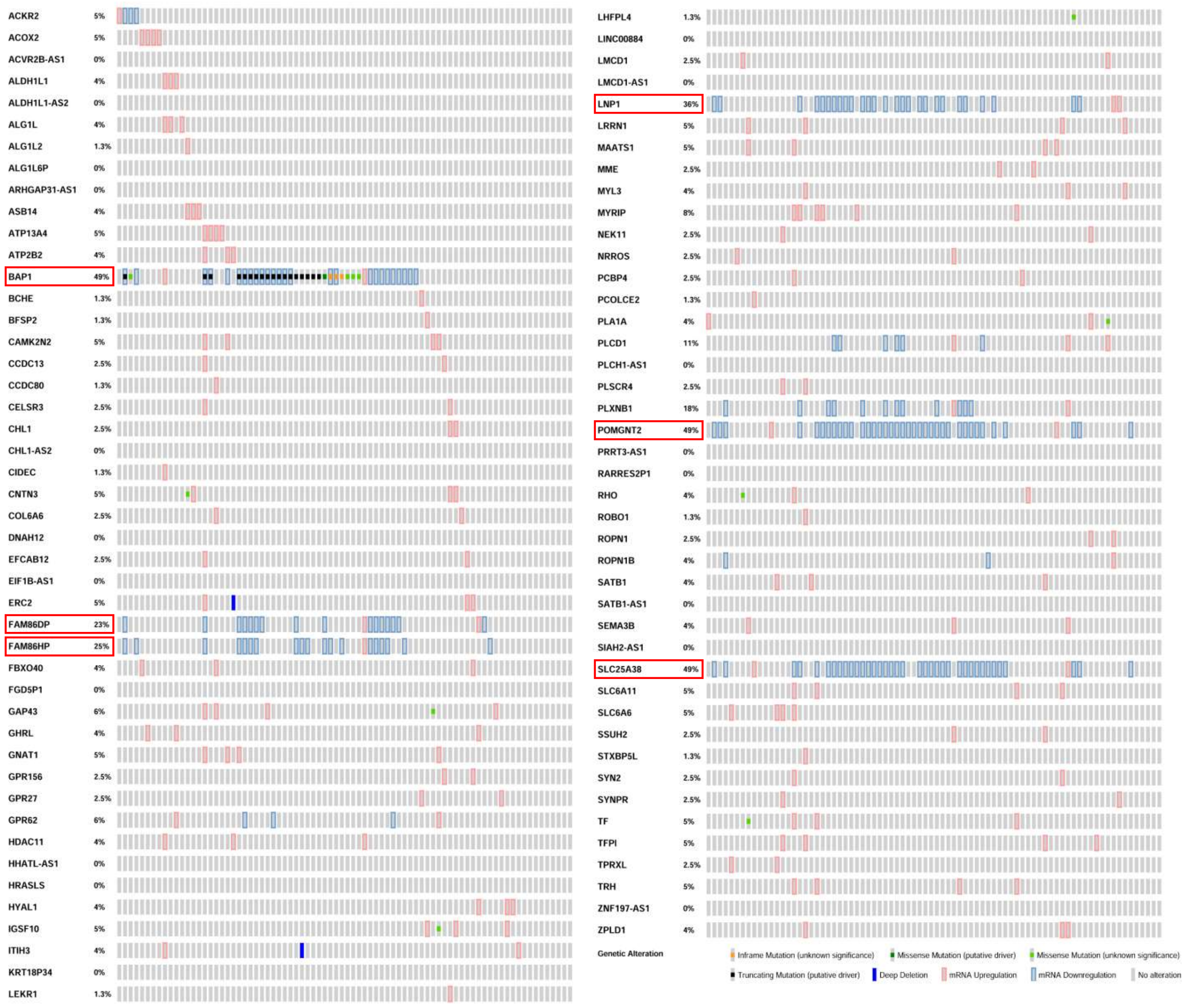

Figure S2. Molecular landscape in 80 primary UMs of TCGA cohort. Mutation and expression status for 89 genes, metastatic status of tumor sample, and chromosome 3 copy number alterations are indicated.

Figure S3

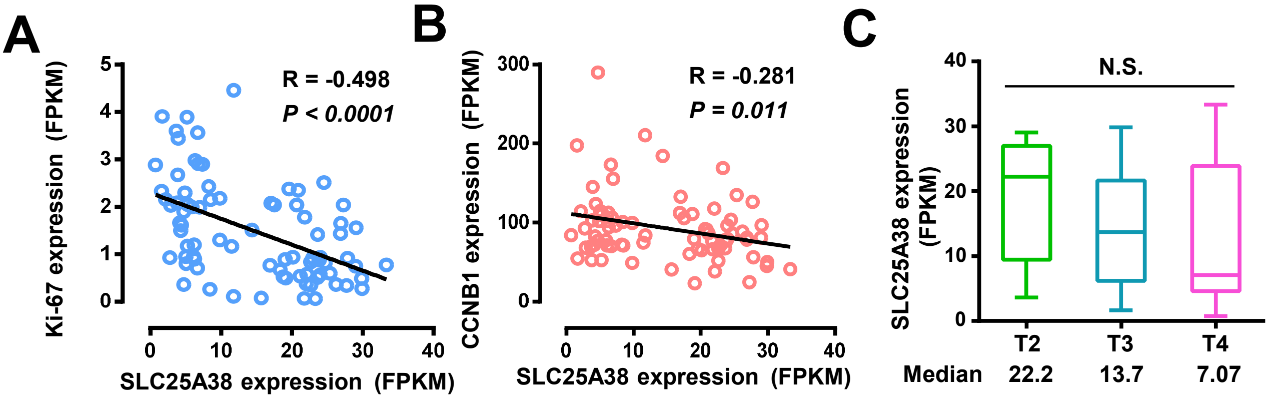

Figure S3. (A-B) Correlation between SLC25A38 expression with Ki-67 expression or CCNB1 expression. Pearson correlation coefficient is indicated. (C) Expression of SLC25A38 in tumors with stage T2 to T4 in TCGA UM cohort. ANOVA with multiple comparisons was used for statistical analysis. Data were obtained from RNA-Seq of TCGA UM data set.

Figure S4

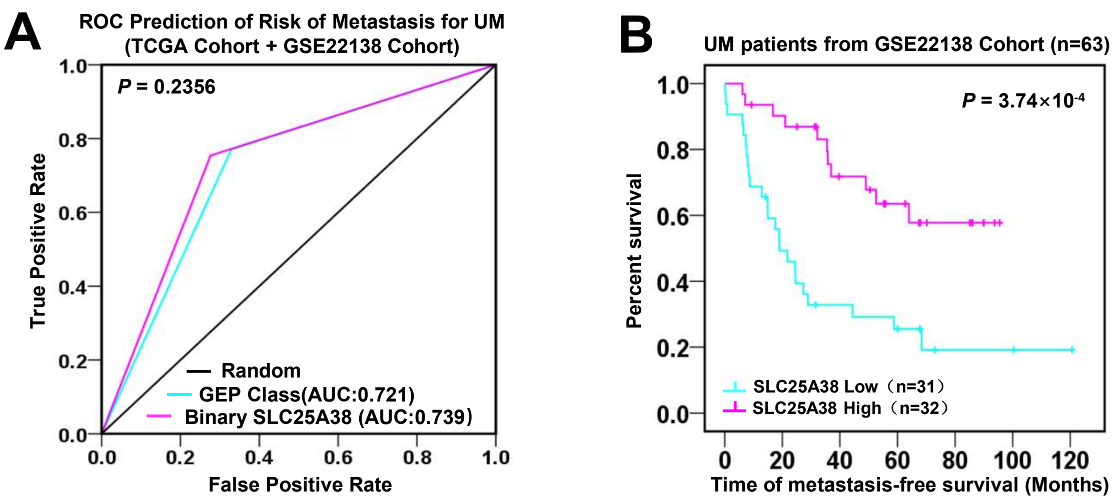

Figure S4. (A) Receiver Operator Curves based on binary SLC25A38 expression and GEP class in all patients containing TCGA cohort and GSE22138 cohort. AUC: Area Under Curve. (B) Kaplan–Meier plots of metastasis-free survival based on SLC25A38 expression level in GSE22138 cohort.

Figure S5

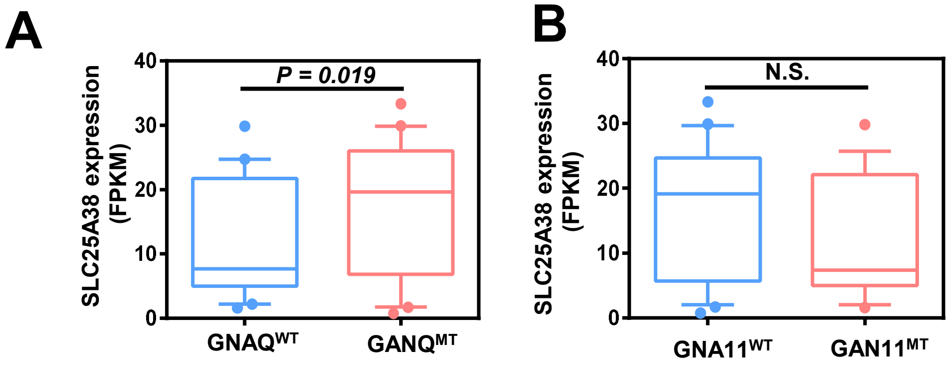

Figure S5. (A-B) Expression of SLC25A38 in tumors with (A) GNAQ and (B) GAN11 mutation status of TCGA UM data set.

Figure S6

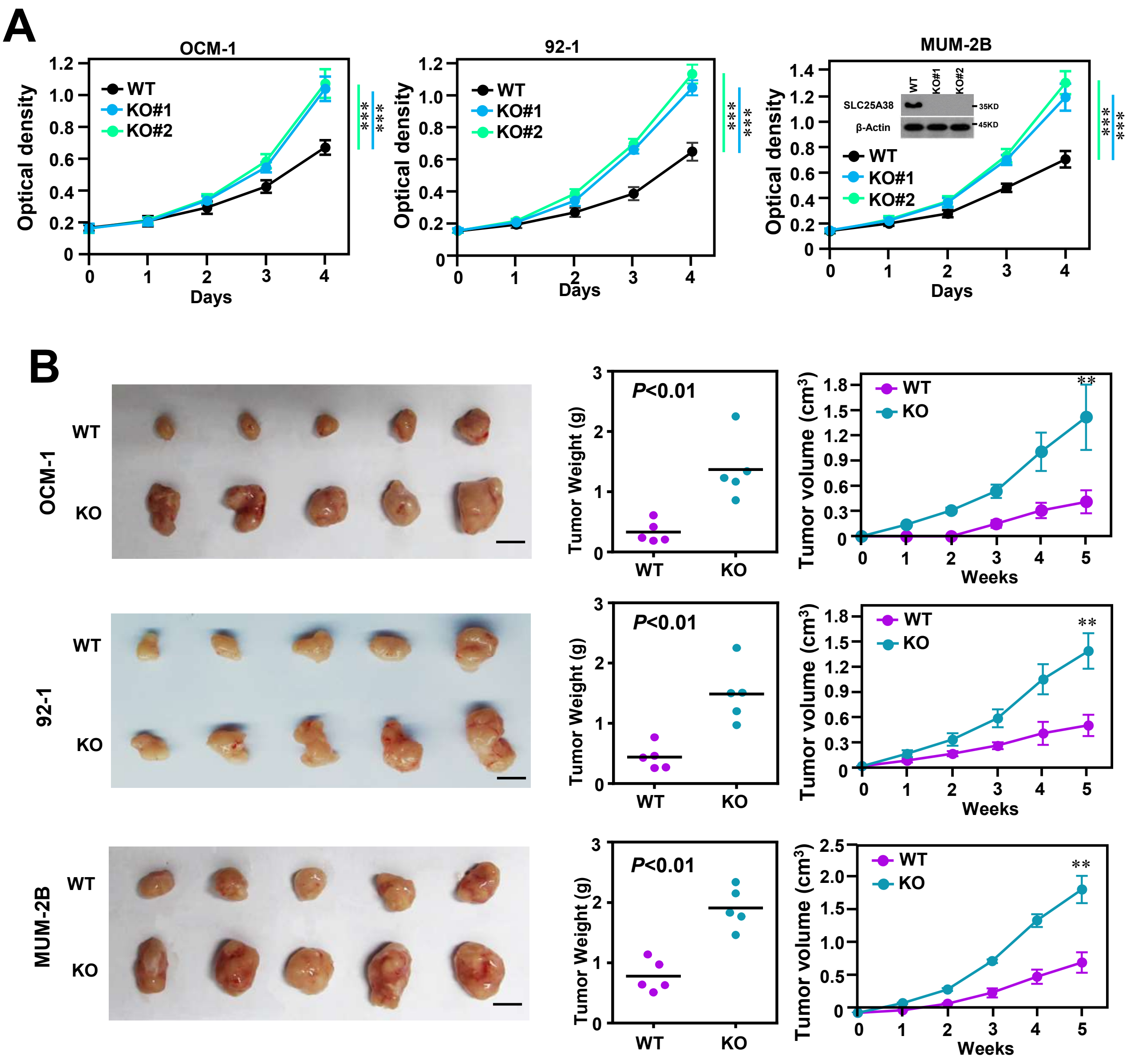

Figure S6. (A) CCK8 assays showing that SLC25A38 knock-out led to increasing proliferation of UM cells. (B) Tumors with SLC25A38 knock-out grew larger and faster in vivo. ** indicates P<0.01.

Figure S7

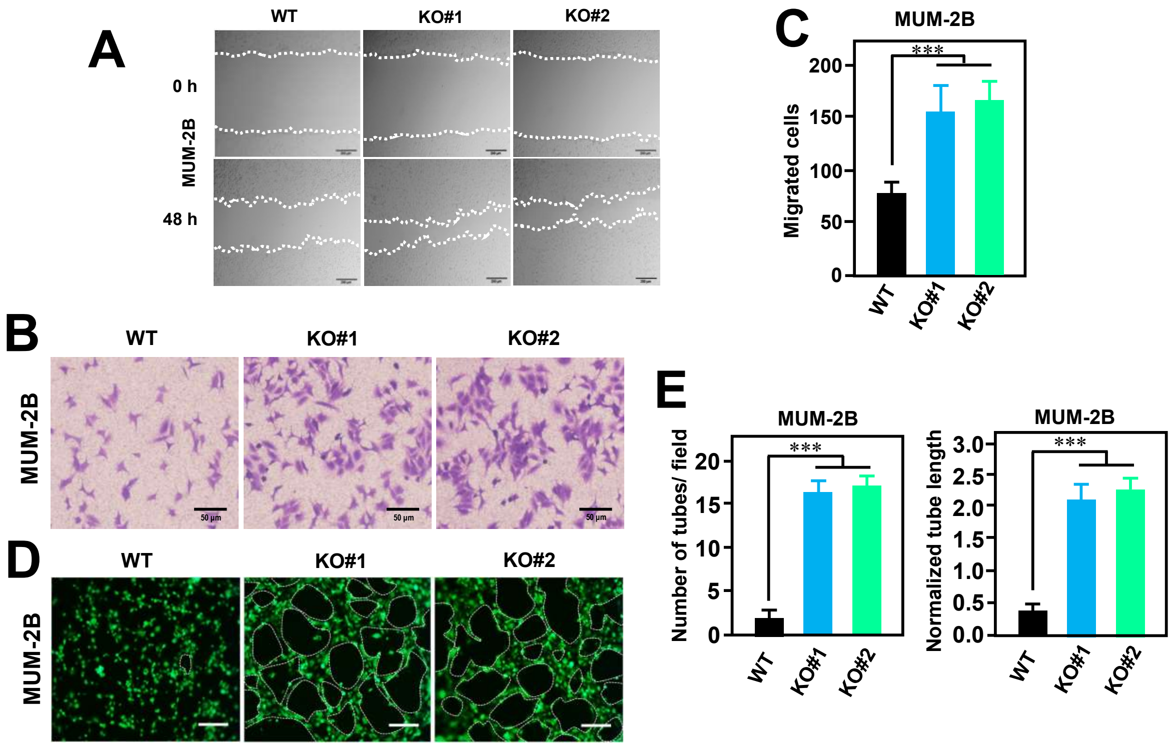

Figure S7. (A-C) Wound healing assay (A) and transwell migration assay (B-C) showing that SLC25A38 knock-out could enhance the migration ability of UM cells. (D-E) Vascular ring formation analyses indicating the increasing ability of angiogenesis upon SLC25A38 knock-out. Number of tubes were counted and normalized tube length were calculated, scale bar 100μm.

Figure S8

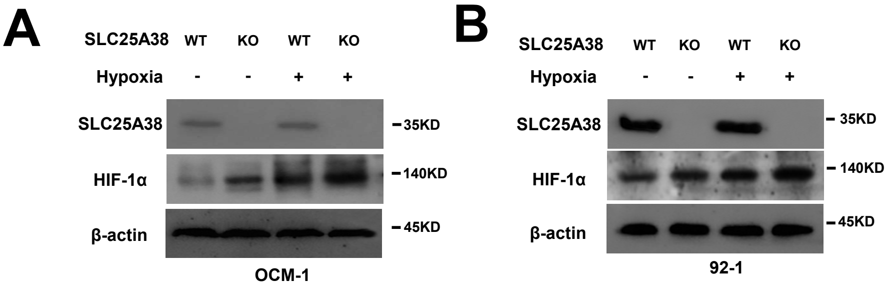

Figure S8. SLC25A38 knock-out could enhance the protein level of HIF-1α in UM cells under conditions of hypoxia.

Figure 4A SLC25A38 of OCM-1

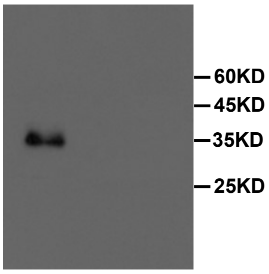

Figure 4A β-Actin of OCM-1

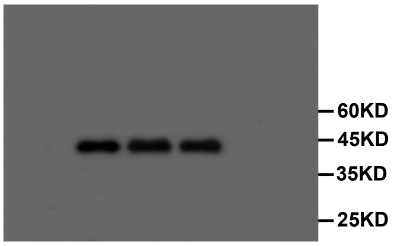

Figure 4A SLC25A38 of 92-1

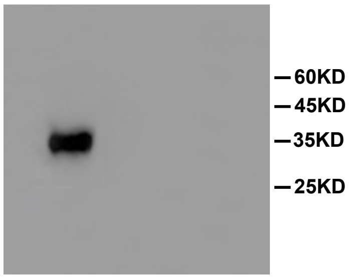

Figure 4A β-Actin of 92-1

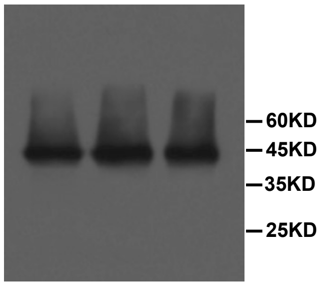

Figure S6A SLC25A38 of MUM-2B

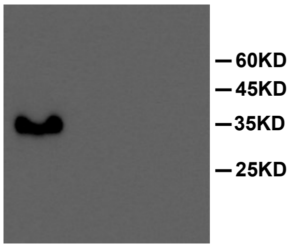

Figure S6A β-Actin of MUM2B

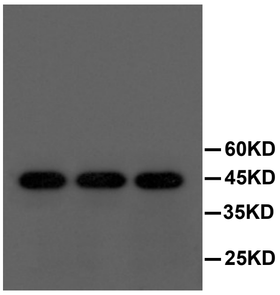

Figure S8A SLC25A38 of OCM-1

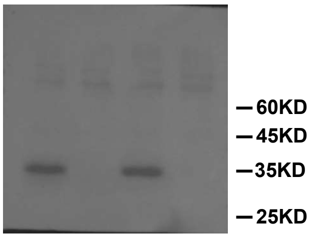

Figure S8A HIF-1α of OCM-1

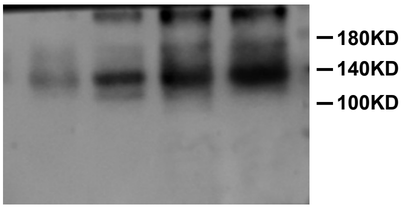

Figure S8A β-Actin of OCM-1

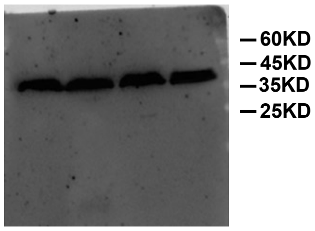

Figure S8B SLC25A38 of 92-1

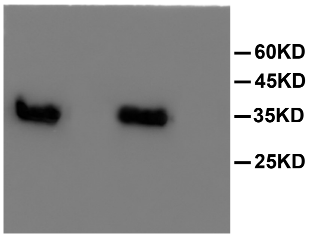

Figure S8A HIF-1α of 92-1

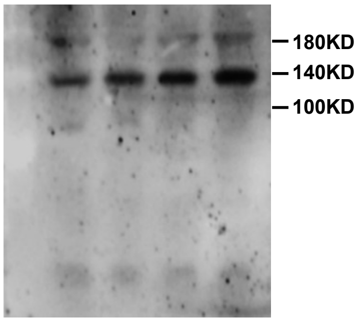

Figure S8A β-Actin of 92-1

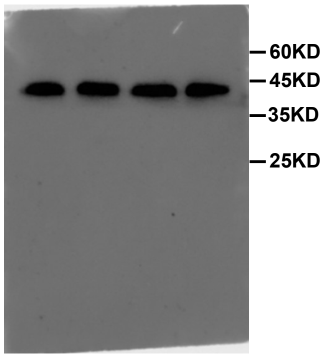

Table S1. Primers used for Real-time PCR

GENE	Forward (5’→3’)	Reverse (5’→3’)
GLP2R	5′ TCACTGATGATCAAGTTGAAGG 3′	5′ TCAGCGCCATCTCCTTCCGAGA 3′
BDKRB2	5′ TGCAGATCATGCAGGTGCTGC 3′	5′ ACCTCCCAAGACTTCTTTCGGA 3′
INHBA	5′ CCTGGACGTTCGGATTGCCTG 3′	5′ CAGATGTTGACCTTGCCATCA 3′
IGFBP5	5′ AGCTGAAGGCTGAAGCAGTGA 3′	5′ GCCACGTTTGCGGCCACGGGA 3′
ATF3	5′ AATAAGATTGCAGCTGCAAAG 3′	5′ ATCTGTTGGATAAAGAGGTT 3′
CDH5	5′ CAGTTTGACCGGGAGCATACC 3′	5′ AGGAAGATGAGCAGGGTGAT 3′
BMP2	5′ GCTGTGATGCGGTGGACTGCA 3′	5′ CACGTCACTGAAGTCCACGTA 3′
AMOT	5′ GCTGGATCAGGCTTGCTCTCC 3′	5′ TCGATTCCGTCCCACGTTCAG 3′
SEMA5A	5′ GAATACCAGGAATGCAACAT 3′	5′ CACACTCAGACCAGTCCGAC 3′
SERPINE1	5′ TTCCACAAATCAGACGGCAGC 3′	5′ AGAACTTGGGCAGAACCAGGA 3′
DDAH1	5′ GGTCTAGTGAATCTGCACAGA 3′	5′ ATTAAAACTGAGCAGCAGGTG 3′
TGFA	5′ GTGTCTGCCATTCTGGGTACG 3′	5′ AGATGAGGGCCCGGCACCACT 3′
FGF8	5′ GTCTTCACGGAGATTGTGCTG 3′	5′ GTTGAGGAACTCGAAGCGCA 3′
PLXDC1	5′ ACTATGGCTGTGCACAGGAGG 3′	5′ CGATGCCCACGATGGTGCCCA 3′
CYR61	5′ CAACCCTGAGTGCCGCCTTGT 3′	5′ CGTGCAGCATCGGCCGTCCAC 3′
BDKRB1	5′ TCTTCAACTACCACATCCTGG 3′	5′ AGGCCCAGGTCAATGAAGTCC 3′
CCL5	5′ CACCCTGCTGCTTTGCCTACA 3′	5′ CTCATCTCCAAAGAGTTGATG 3′
HDAC9	5′ CGCACACACGTGCGCTCTCTG 3′	5′ TACTCTGTATTCGTCCAGCAT 3′
ID1	5′ CAGGTAAACGTGCTGCTCTAC 3′	5′ ATCTCGCCGT TGAGGGTGCT 3′
PSG2	5′ GTCACAAAGTATACTGCAGGA 3′	5′ TGTCCTGATTGCTGAAACTTC 3′
ARHGDIB	5′ ACTGGAGATCTGGAAGCCCTC 3′	5′ ACTCATACTCCTCAGGCCGA 3′
CLSTN2	5′ GCTTCATAACTTAGATTTCTG 3′	5′ ATGTAGCACCTGCTCATAGC 3′
FERMT3	5′ GATCTACCTGCGGTGCCAGGA 3′	5′ TGGCCTTGAACTTTCGCTGGA 3′
FLRT1	5′ AATGCCTACGTAGCTGATGAG 3′	5′ GCAGCTCGCCAGCCTGGTGCA 3′
SRPX	5′ CCACCTGTGAGTTCTCCTGCA 3′	5′ ATTCCTAGCTGGAGCCGGTAA 3′
CADM4	5′ AGCAAGCAGACGCAGTACGTG 3′	5′ GCCGTTATCCGCGGATACCAG 3′
MADCAM1	5′ CCGACACCACCTCCCAGGAGC 3′	5′ ACAGAGCCGCGGGCAGCTGGT 3′
CLDN3	5′ CAGGCGTGCTGTTCCTTCTCG 3′	5′ TGGCCGTGTACTTCTTCTCGC 3′
CEACAM19	5′ CCTGCTCCGAGGGGCCAGGGA 3′	5′ TATGGACCTTGTGTCACTGAT 3′
FGF12	5′ TATGTGGCCATGAATGGTGAA 3′	5′ TACAAAATGTGATGAGGGCTT 3′
HIF1A	5′ AATATTGATTGCATCTCCATC 3′	5′ ATCTTTGGATTTAGTTCTTCC 3′
CBP	5′ CGTCAAAGTGTTAATGTAGAG 3′	5′ TTCTGTGAGAGTGGGGTCTTC 3′
EID3	5′ AGAAGTTGGACCTGAGTAGTT 3′	5′ GCCTGTCTTCATCAAGCCTTA 3′
β-actin	5′ ATCACCATTGGCAATGAGCG 3′	5′ TTGAAGGTAGTTTCGTGGAT 3′

Table S2. Clinicopathologic Features of Patients with Uveal Melanoma without and with Metastasis from TCGA

Terms	No metastasis (n=54)	Metastasis (n=26)	*P* Value
**Age（years）**	61.69 (13.83)	61.58 (14.46)	0.9743
**Tumor Basal Diameter (mm)**	16.39 (3.494)	18.096 (3.177)	**0.0408**
**Tumor Thickness (mm)**	9.994 (2.821)	11.308 (2.617)	**0.0495**
**Gender**			
Male	30	15	0.857
Female	24	11	
**Clinical Stage**			
Stage II	29	10	0.174
Stage III、IV	24	16	
**Tumor Location**			
Choroid	39	18	0.782
Choroid|Ciliary body	15	8	
**Chromosome 1 loss**			
NO	49	22	0.417
YES	5	4	
**Chromosome 3 loss**			
NO	38	11	**0.016**
YES	16	15	
**Chromosome 6p gain**			
NO	37	20	0.437
YES	17	6	
**Chromosome 8q gain**			
NO	36	11	**0.038**
YES	18	15	

Table S3. Univariate and Multivariate Analysis of Various Clinical Parameters for OS in Patients with UM by Cox-Regression Analysis from TCGA Dataset

Terms	Univariate analysis		Multivariate analysis	
	**HR（95%CI）**	***P* value**	**HR（95%CI）**	***P* value**
**Age**（years）				
≤Median	1.00		1.00	
>Median	2.126(0.915-4.938)	**0.080**	2.914(1.116-7.283)	**0.022**
**Gender**				
Male	1.00			
Female	0.649(0.274-1.538)	0.327	—	NA*
**Clinical Stage**				
Stage II	1.00			
Stage III+IV	1.500(0.628-3.580)	0.361	—	NA*
**Tumor Basal Diameter (mm)**				
≤Median	1.00		1.00	
>Median	2.390(0.991-5.761)	**0.052**	1.557(0.589-4.118)	0.372
**Tumor Thickness (mm)**				
≤Median	1.00			
>Median	1.641(0.703-3.834)	0.252	—	NA*
**Tumor Location**				
Choroid	1.00			
Choroid|Ciliary body	1.004(0.409-2.462)	0.994	—	NA*
**Chromosome 1 loss**				
No	1.00			
Yes	0.333(0.045-2.483)	0.284	—	NA*
**Chromosome 3 loss**				
No	1.00		1.00	
Yes	2.836(1.206-6.673)	**0.017**	2.906(0.872-9.680)	0.082
**Chromosome 6p gain**				
No	1.00			
Yes	0.371(0.109-1.260)	0.112	—	NA*
**Chromosome 8q gain**				
Low	1.00		1.00	
High	2.241(0.931-5.392)	**0.072**	1.319(0.388-4.483)	0.658

Bold *P* value indicates statistical significance.

*** Not assessed due to an insignificant result in the univariate analysis (P > 0.1).**

Table S4. Univariate and Multivariate Analysis of Gene Change for OS in Patients with UM by Cox-Regression Analysis from TCGA Dataset

Variable	Univariate Logistic Regression		Multivariate Logistic Regression	
	HR（95%CI）	*P* value	HR（95%CI）	*P* value
**BAP1 Low Expression**				
No	1.00		1.00	
Yes	3.217(1.407-7.354)	**0.006**	0.381(0.126-1.150)	0.087
**FAM86DP Low Expression**				
No	1.00		1.00	
Yes	3.492(1.474-8.273)	**0.004**	1.870(0.644-5.434)	0.250
**FAM86HP Low Expression**				
No	1.00		1.00	
Yes	2.849(1.211-6.701)	**0.016**	0.796(0.257-2.463)	0.693
**LNP1 Low Expression**				
No	1.00		1.00	
Yes	4.072(1.725-9.613)	**0.001**	0.628(0.204-1.938)	0.419
**POMGNT2 Low Expression**				
No	1.00		1.00	
Yes	9.412(3.117-28.42)	**7×10^{-5}**	3.200(0.534-19.16)	0.203
**SLC25A38 Low Expression**				
No	1.00		1.00	
Yes	18.017(4.205-77.21)	**9.8×10^{-5}**	45.58(4.599-451.6)	**0.001**
**BAP1 Mutation**				
No	1.00		1.00	
Yes	2.923(1.280-6.676)	**0.011**	0.533(0.198-1.436)	0.213
**Chromosome 3 loss**				
No	1.00		1.00	
Yes	2.836(1.206-6.673)	**0.017**	0.365(0.112-1.192)	0.095

Bold *P* value indicates statistical significance.

Table S5. Univariate and Multivariate Analysis of Various Clinical Parameters for OS in Patients with UM by Cox-Regression Analysis from TCGA Dataset

Terms	Univariate analysis		Multivariate analysis	
	**HR（95%CI）**	***P* value**	**HR（95%CI）**	***P* value**
**Age**（years）				
≤Median	1.00		1.00	
>Median	2.126(0.915-4.938)	**0.080**	4.134(1.636-10.445)	**0.003**
**Gender**				
Male	1.00			
Female	0.649(0.274-1.538)	0.327	—	NA*
**Clinical Stage**				
Stage II	1.00			
Stage III+IV	1.500(0.628-3.580)	0.361	—	NA*
**Tumor Basal Diameter (mm)**				
≤Median	1.00		1.00	
>Median	2.390(0.991-5.761)	**0.052**	2.508(0.941-6.684)	0.066
**Tumor Thickness (mm)**				
≤Median	1.00			
>Median	1.641(0.703-3.834)	0.252	—	NA*
**Tumor Location**				
Choroid	1.00			
Choroid|Ciliary body	1.004(0.409-2.462)	0.994	—	NA*
**SLC25A38 Low Expression**				
No	1.00		1.00	
Yes	18.017(4.205-77.21)	**9.8×10^{-5}**	23.90(5.454-104.7)	**2.6×10^{-5}**

Bold *P* value indicates statistical significance.

*** Not assessed due to an insignificant result in the univariate analysis (*P* > 0.1).**
